# Exciton-to-Trion Conversion in Monolayer WS_2_ under Pressure

**DOI:** 10.1021/acs.nanolett.5c02823

**Published:** 2025-08-29

**Authors:** Beatrice D’Alò, Mattia Capeccia, Lilia Boeri, Paolo Postorino, Elena Stellino

**Affiliations:** † Department of Physics, 9311 Sapienza University of Rome, Piazzale Aldo Moro 5, Rome 00185, Italy; ‡ Department of Basic and Applied Sciences for Engineering, Sapienza University of Rome, Piazzale Aldo Moro 5, Rome 00185, Italy

**Keywords:** two-dimensional semiconductors, monolayer WS_2_, exciton-to-trion conversion, high pressure, photoluminescence spectroscopy

## Abstract

Exciton-to-trion conversion in two-dimensional semiconductors defines the transition from an optoelectronics based on neutral bosons to one based on charged fermions, with a huge impact on the transport and spin/valley-related properties. This process has been successfully induced in field-effect transistors under gate voltage, chemically doped samples, and nonuniformly nanoscale-strained materials. Here, we study the evolution of the photoluminescence spectrum of monolayer WS_2_ under high pressure, decoupling exciton and trion contributions by their responses to laser-power variations. We demonstrate that crystal compression drives a substrate-independent, partially reversible exciton-to-trion conversion, with trion recombination dominating the emission above 3 GPa. The observed mechanism does not rely on external charge injection but involves the pressure evolution of intrinsic doping levels within the band structure. Our results indicate that trion-based emission can be achieved by reshaping the periodic crystal potential via the modulation of interatomic interactions, offering a novel approach to the study of exciton-to-trion conversion in two-dimensional materials.

## Graphical abstract

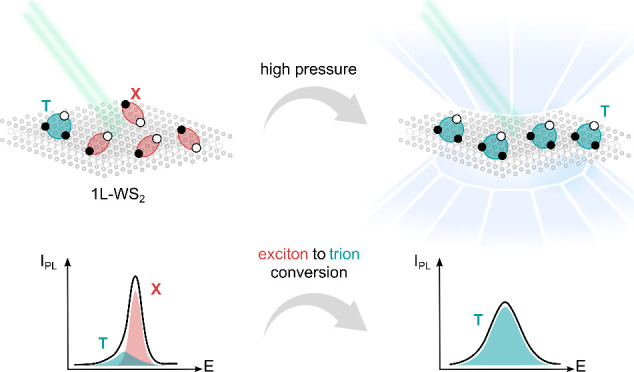

Excitons dominate the optoelectronic response of two-dimensional (2D) semiconductors, where in-plane confinement reduces dielectric screening and enhances the Coulomb interaction between electron–hole pairs.^1^ When additional charge carriers are introduced in the crystal, excitons can interact with residual free electrons and holes to form negative and positive trions,^2^ which are three-body quasiparticles considered as the semiconductor equivalent of H^–^ and H_2_^+^.

Recently, there has been growing interest in understanding the mechanisms governing exciton-to-trion conversion, which stands as a pivotal process for many aspects of the physics of 2D materials.

The net charge of trions enables them to acquire a nonzero drift velocity in an external electric field, in contrast to the insensitivity of neutral excitons, with a huge impact on the transport properties of the sample.^3,4^ In this framework, an efficient manipulation of trions can potentially lead to the design of (charged) exciton-based optoelectronic devices with operation principles similar to those of conventional electronic devices.

The fermionic nature of trions was also found to deeply influence the degree of valley polarization in transition-metal dichalcogenide (TMD) semiconductors.^5,6^ In monolayer (1L) TMDs, electronic excitation and recombination at the inequivalent valleys, *K* – *K*′, are selectively activated by circularly polarized light with opposite helicity. However, intervalley exchange interaction, mediated by phonons, degrades the valley polarization leading to largely depolarized radiative recombination at ambient conditions. In this process, it was demonstrated that conversion of electron–hole pairs into trions protects valley polarization, as the intervalley exchange of trions would require the spin flip of individual carriers, which is energetically and spin forbidden.^7^

To date, efficient exciton-to-trion conversion in 2D semiconductors (typically TMDs) has been induced using a variety of means. The most straightforward approach consists in directly introducing extra charge carriers into the sample by applying a gate voltage^4,8–10^ or synthesizing chemically doped materials.^5,11^ Less intuitively, sharp modifications of the crystal morphology have also been found to promote trion formation.^12–14^ Highly nonuniform strain gradients, localized at the nanoscale, can indeed facilitate the funneling of both excitons and free electrons toward regions of maximum strain.^12–14^ In this configuration, spatial confinement enhances exciton-electron binding, leading to an increase in trion population.^12,13^

In our work, we conduct a thorough investigation of the pressure evolution of the photoluminescence (PL) spectrum of 1L-WS_2_, decoupling exciton and trion contributions by their different responses to variations of the incident laser power. We demonstrate that the compression of the 1L-WS_2_ crystal drives a substrate-independent, partially reversible exciton-to-trion conversion process, with the trion recombination dominating the sample emission already above 3 GPa. Compared with approaches based on chemical doping and gate voltage application, the mechanism here observed does not rely on the injection of extra charge carriers in the sample, but it is based on the pressure evolution of the intrinsic doping levels within the electronic band structure. Moreover, in contrast with the conversion processes locally activated in nonuniformly strained flakes, the pressure-induced increase in the trion population occurs at the crystal scale.

High-pressure, power-dependent PL measurements were performed on 1L-WS_2_ crystals deposited on bulk hexagonal boron nitride (1L-WS_2_/hBN) and on a culet of a diamond anvil cell (1L-WS_2_/diamond). Both 1L-WS_2_ and bulk hBN were mechanically exfoliated from the corresponding millimeter-sized crystals purchased by HQ graphene. 1L-WS_2_ flakes were identified by micro-Raman and PL measurements.^15–18^ Both 1L-WS_2_/hBN and 1L-WS_2_/diamond systems were assembled by a deterministic dry transfer method^19^ using a 2D-transfer system by HQ graphene.

A screw-driven diamond anvil cell (DAC) equipped with type IIa diamonds with 600-μm-diameter culets was employed for pressure application. Diamonds were separated by a stainless steel gasket, in which a 250-μm-diameter hole was drilled as a measurement chamber. A 4:1 methanol–ethanol mixture was used as pressure transmitting medium, providing hydrostatic conditions up to 10 GPa.^20^ A micrometric ruby sphere was loaded in the DAC as pressure gauge.^21^ Pressure was increased in small steps from 0 to 8 GPa (we recall that hBN remains isostructural up to 10 GPa^22,23^ ).

PL measurements were performed by a Horiba LabRam HR Evolution microspectrometer coupled with a grating monochromator with 600 grooves/mm and a Peltier-cooled charge-coupled-device detector.^24^ A 532 nm solid-state laser was employed as a light source. The laser spot was focused on the sample surface by a 50× objective, resulting in a spot size of ∼2.5 μm in diameter. Neutral-density filters with different optical densities were used to vary the laser power in the 0.01–2.5 mW range, as measured at the laser outlet. The effective laser power impinging on the sample was estimated to be reduced by ∼40% due to the presence of losses in the optical path.

To rule out spatial variations in the pressure response, we measured 1L-WS_2_/hBN and 1L-WS_2_/diamond at four different positions, obtaining analogous pressure trends, as shown in section S1 of the Supporting Information.

At ambient conditions, the PL spectrum of 1L-WS_2_ exhibits an intense peak at ∼2.0 eV, ascribed to the radiative recombination of the A exciton^14^ across the direct band gap, and a lower-energy shoulder at ∼1.97 eV, ascribed to the recombination of the negative trion T; see Figure 1f (all panels of Figure 1 show PL spectra of 1L-WS_2_/hBN at selected pressure values; the complete data sets for both 1L-WS_2_/hBN and 1L-WS_2_/diamond are reported in sections S3 and S4 of the Supporting Information). The intrinsic nature of the trion quasiparticle makes the T band a spectroscopic signature for the presence of excess charge carriers.^9,25,26^ In semiconducting TMDs, these typically arise from chalcogen vacancies or impurities intercalated during the growth process, which produce a mild n-type doping with charge carrier density of the order of ∼10^14^–10^15^ cm^–3^.^27–29^

**Figure 1:**
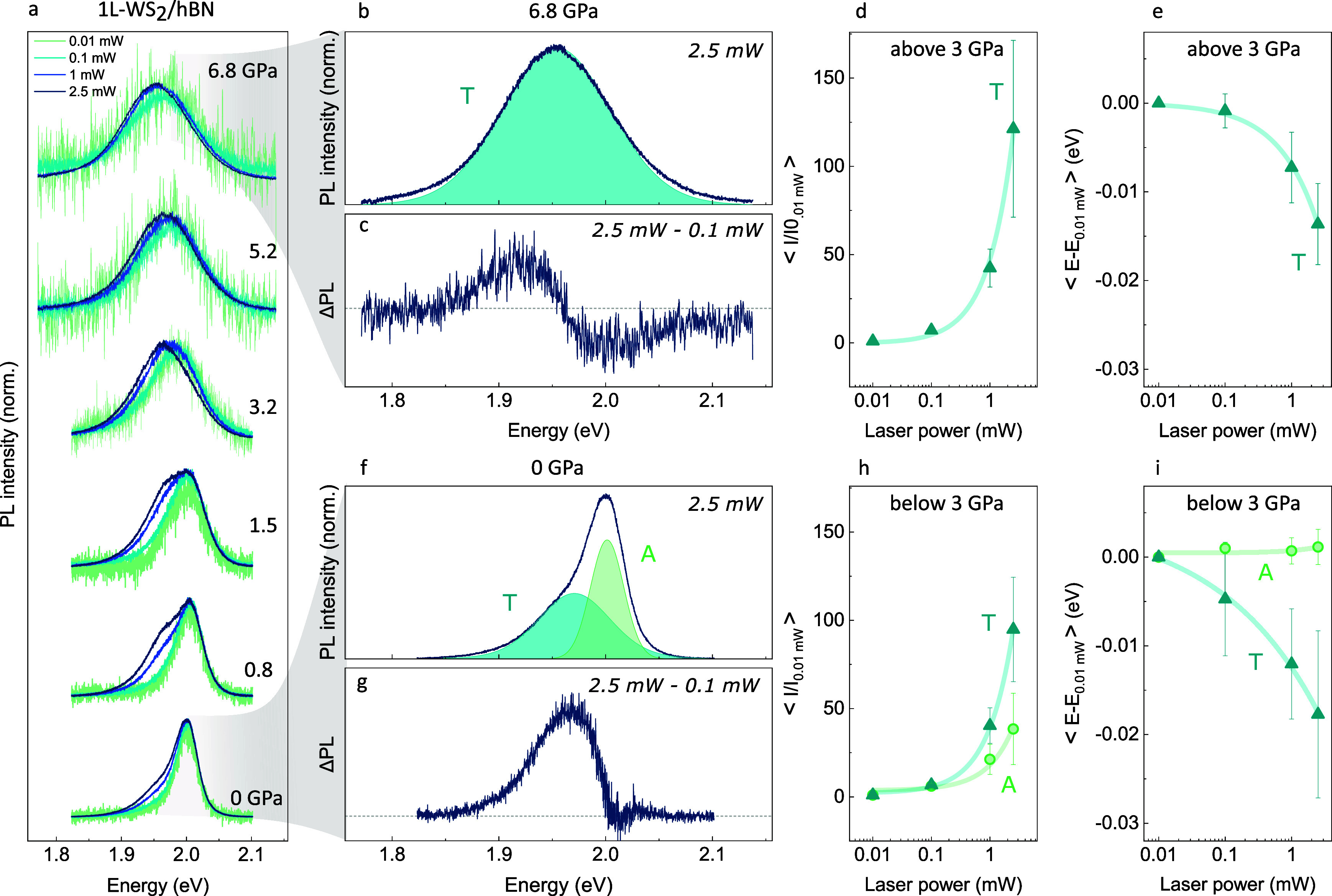
(a) Normalized high-pressure PL spectra of 1L-WS_2_/hBN collected at different laser powers. The absolute PL intensities versus pressure for each laser power are reported in section S2 of the Supporting Information. (b, f)­PL spectra of 1L-WS_2_/hBN at 2.5 mW collected at 6.8 GPa (b) and 0 GPa (f); the best-fit curves for the A exciton and T trion are shown in green and cyan, respectively. (c, g) Difference (ΔPL) between the PL spectra at 2.5 and 0.1 mW, normalized to their maximum, collected at 6.8 GPa (c) and 0 GPa (g). (h, d) Power dependence of the peak intensity in the low (h) and high (d) pressure regimes. Blue triangles represent the mean trion area, normalized to the 0.01 mW value, upon increasing the laser power: ⟨*I*/*I*_0.01 mW_⟩. Each data point is obtained by averaging *I*/*I*_0.01 mW_ at a given laser power over all measurements in the 0.0–2.9 GPa (h) and 3.2–6.8 GPa (d) ranges. Error bars indicate the associated standard deviations. Green circles in panel­(h) represent the ⟨*I*/*I*_0.01 mW_⟩ values for the A exciton peak in the 0.0–2.9 GPa range, with the corresponding error bars. (i, e) Power dependence of the peak positions in the low (i) and high (e) pressure regimes. Blue triangles represent the mean energy shift of the trion center, relative to the 0.01 mW position upon increasing the laser power: ⟨*E* – *E*_0.01 mW_⟩. Each data point is obtained by averaging *E* – *E*_0.01 mW_ at a given laser power over all measurements in the 0.0–2.9 GPa (i), 3.2–6.8 GPa (e) ranges. Error bars indicate the associated standard deviations. Green circles in panel (i) represent the ⟨*E* – *E*_0.01 mW_⟩ values for the A exciton in the 0.0–2.9 GPa range, with the corresponding error bars.

In the PL process, the probability of forming excitonic or trionic states is correlated with the density of photoexcited electrons in the conduction band, leading to a strong laser power dependence of the A-to-T relative intensity.^9,26,30,31^ As shown in Figure 1h, upon increasing the laser power from 0.01 to 2.5 mW, the growth rate of the trion intensity is more than 2 times larger compared to that of the exciton intensity. Moreover, the T peak undergoes a progressive redshift as a function of the laser power, while the A band remains fixed in energy; see Figure 1i.^26,30,31^

The different responses of A and T peaks to laser power variations can be exploited as a benchmark to decouple the evolution of excitons and trions in PL spectra at high pressures. Figure 1a shows the combined pressure and power dependence of selected PL spectra in the 0.01–2.5 mW and 0–7 GPa ranges, normalized to the maximum of each curve. In the low-pressure regime, below 3 GPa, as the laser power increases at fixed pressure, the spectrum evolves from a single-peaked to a double-peaked profile, with the trion feature becoming progressively more evident on the low-energy side of the main exciton band; see Figure 1a.

Above 3 GPa, the power-dependent spectral response visibly changes: at each pressure, the spectrum maintains an identical (quasi) symmetric profile upon increasing the laser power, suggesting the presence of a unique recombination channel in the PL signal. An example is shown in Figure 1b, where the PL spectrum at 6.8 GPa displays a perfectly single-peaked profile at the maximum power of 2.5 mW. The power evolution of this band in terms of intensity and energy, reported in Figures 1d,e, respectively, clearly indicates the fully trionic nature of the PL spectrum in the high-pressure regime.

To further compare the power dependence of the PL signal in the low-pressure and high-pressure regimes, panels g and c of Figure 1 show the difference between the normalized spectra at 2.5 and 0.1 mW (at fixed pressure) for 0 and 6.8 GPa, respectively: ΔPL­(*E*)|_P_ = [PL_2.5_(*E*) – PL_0.1_(*E*)]_P_. In Figure 1g, the peaked profile of ΔPL­(*E*)|_0_ suggests that, at low pressure, the dominant effect of increasing the laser power is a spectral weight transfer from a high-energy (exciton) contribution to a low-energy (trion) contribution. In contrast, in Figure[Fig fig1]c, the interference-like profile of ΔPL­(*E*)|_6.8_ indicates that, at high pressure, the primary effect of increasing the laser power is the shift of a single contribution (trion) toward lower energies.

The detailed evolution of A and T peaks in the 0–8 GPa range, at 1 mW, is shown in Figure 2a,b for 1L-WS_2_/hBN and 1L-WS_2_/diamond, respectively. The best-fit curves and the fitting parameters, reported in panels a, b and c, d of Figure 2, respectively, are obtained, at each pressure, through a global fit procedure over the power-dependent spectra described in section S5 of the Supporting Information.

**Figure 2:**
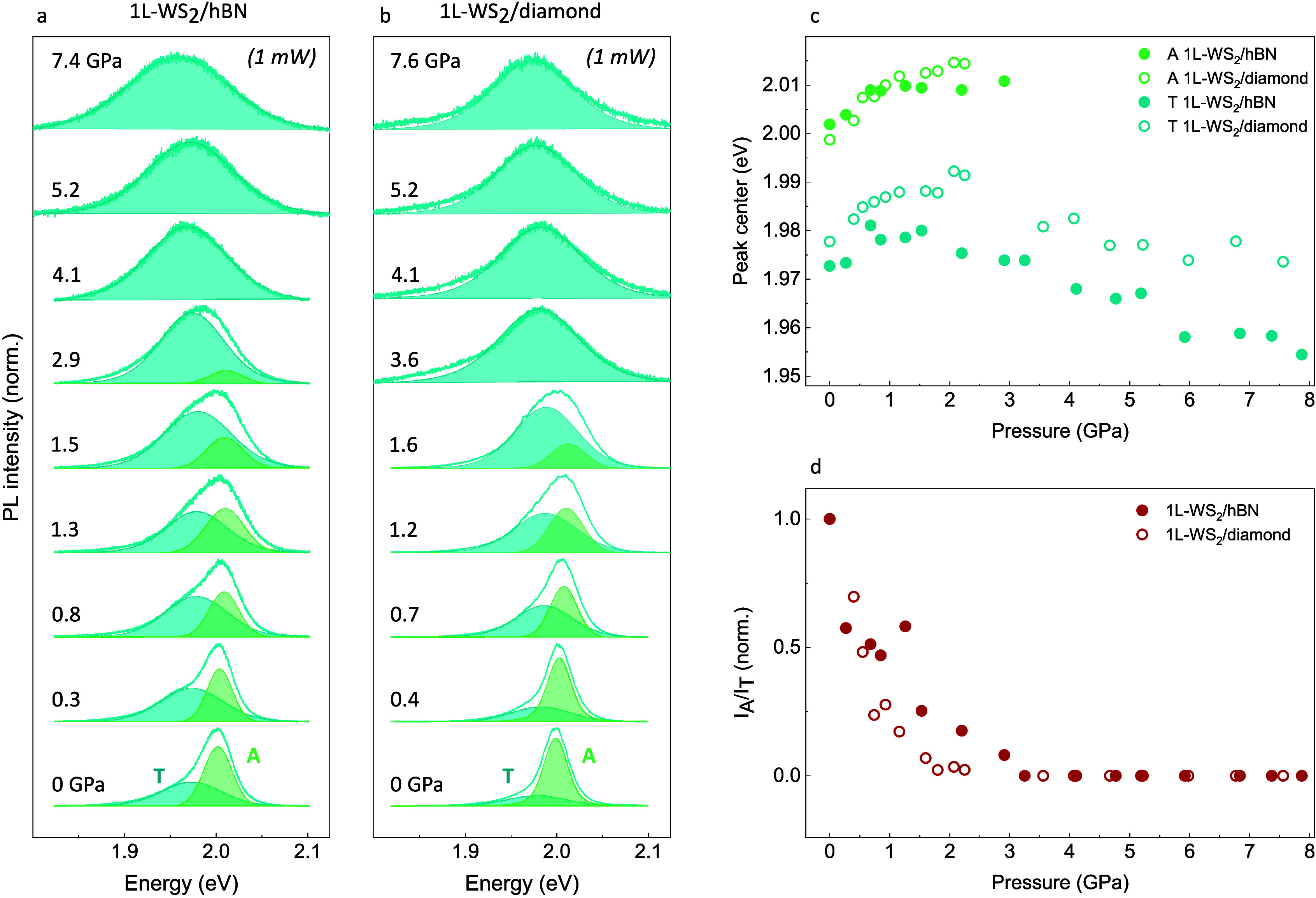
High-pressure PL spectra of 1L-WS_2_/hBN (a) and 1L-WS_2_/diamond (b) collected at 1 mW and normalized to their maximum. The best-fit curves of the A and T peaks are shown in green and cyan, respectively. The curves were obtained by the fitting procedure described in section S5 of the Supporting Information. (c) High-pressure evolution of the A (green) and T (cyan) peak centers in the 1L-WS_2_/hBN (full dots) and 1L-WS_2_/diamond (empty dots) samples measured at 1 mW. (d) High-pressure trends of the exciton-to-trion intensity ratio *I*_A_/*I*_T_ for the 1L-WS_2_/hBN (full dots) and 1L-WS_2_/diamond (empty dots) samples collected at 1 mW, normalized to their value at 0 GPa. *I*_A_ and *I*_T_ were obtained from the fit of the PL spectra, in which *I*_A_ = 0 above 3 GPa.

In the 0–3 GPa range, both exciton and trion energies display a growing sublinear trend, indicating an increase in the band-gap energy in agreement with experimental and theoretical literature.^32–34^ At the same time, a progressive transfer of spectral weight is observed from A to T, until the exciton peak becomes indistinguishable in the spectrum above ∼3 GPa in both 1L-WS_2_/hBN and 1L-WS_2_/diamond. Figure 2d shows the pressure trend of the A-to-T intensity ratio *I*_A_/*I*_T_ in these two cases.

Above 3 GPa, when the trion recombination remains the sole contribution to the PL signal, the blueshift trend of the T peak is reversed and the band continuously redshifts up to the maximum measured pressure.

Once the pressure on the sample is released, the exciton contribution becomes visible again in the spectrum, and both the A and T bands return to their original energy positions, as shown in Figure 3b,c. Moreover, a decrease in the A-to-T relative intensity is evident, particularly in 1L-WS_2_/diamond, possibly due to the creation of defects during sample compression.

**Figure 3:**
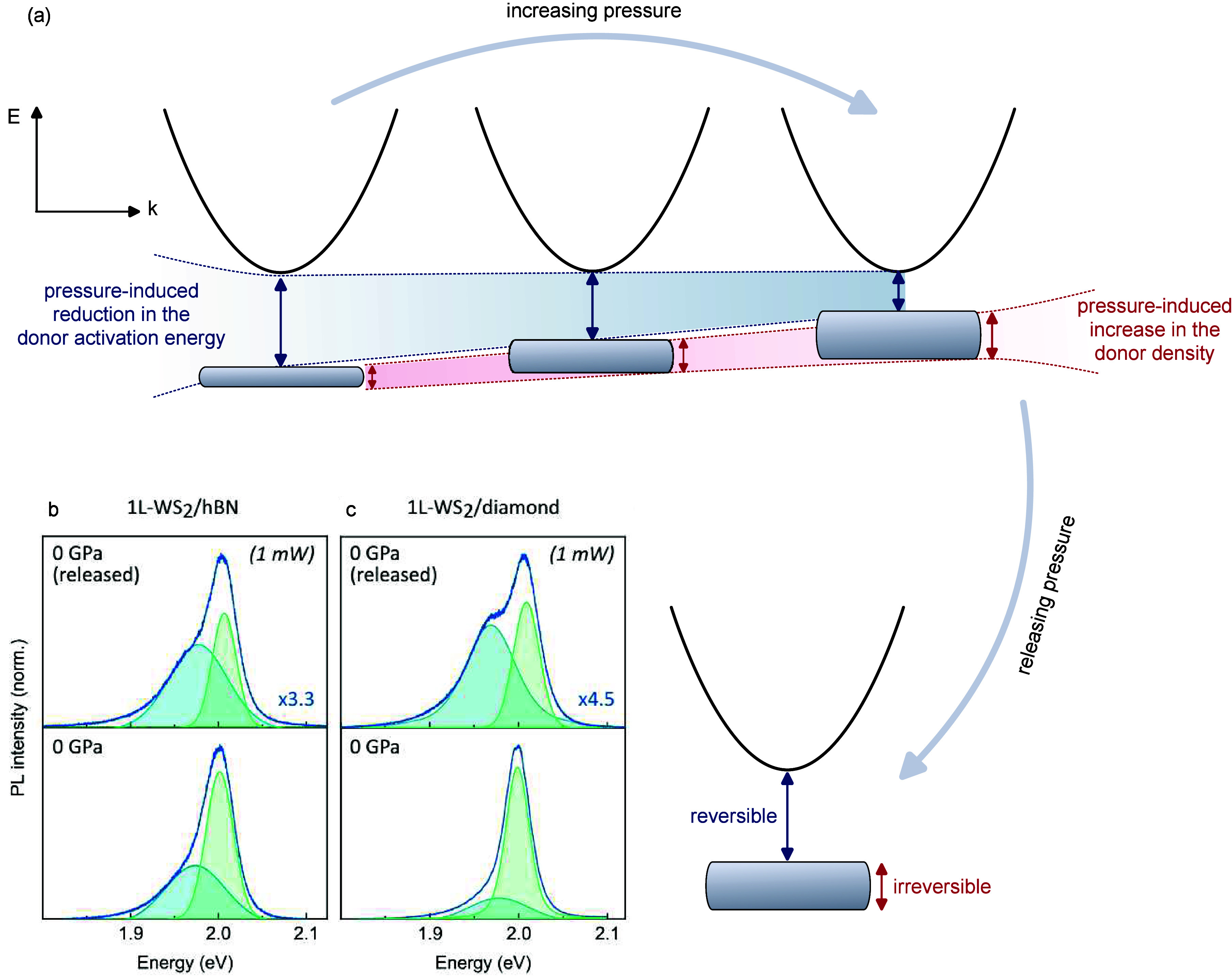
(a) Scheme of the mechanism underlying exciton-to-trion conversion in 1L-WS_2_ discussed in the text. Pressure induces a moderate increase in the density of donors and a progressive reduction in the donor activation energy. The former effect is irreversible, while the latter is reversible once the sample is brought back at ambient conditions. (b, c) PL spectra of 1L-WS_2_/hBN (b) and 1L-WS_2_/diamond (c) before (0 GPa) and after (0 GPa, released) the pressure cycle. All of the spectra were collected at 1 mW. Comparisons at 0.01, 0.1, and 2.5 mW are reported in section S6 of the Supporting Information.

It is worth highlighting the parallelism in the pressure evolution of 1L-WS_2_ crystals deposited on hBN and diamond, as witnessed by the same pressure threshold observed in both cases for the suppression of the exciton feature and the inversion of the trion energy trend. This strong similarity suggests a substantial independence of the pressure response of 1L-WS_2_ from the nature of the substrate. We recall that hBN and diamond present quite different surface properties in their interaction with the overlayer: hBN is inert and atomically flat, whereas diamond typically exhibits reactive dangling bonds in the out-of-plane direction, due to the nature of its sp^3^-hybridized carbon atoms.^29,35–38^

The phenomenology unveiled by coupled pressure- and power-dependent measurements is governed by an efficient exciton-to-trion conversion process occurring within the first 3 GPa. This mechanism represents the dominant effect induced by the application of pressure, while the variation in the band-gap energy, as reflected in the trend of the A exciton, is small in the 0–3 GPa range, and practically unaccounted for in the spectral response at higher pressures.

It is remarkable that the evolution of the 1L-WS_2_ PL spectrum under pressure exhibits a clear analogy with the exciton-to-trion conversion observed in gate-voltage PL experiments.^4,9,26,39^ In the latter, the application of voltage on field-effect-transistor-structured 1L-TMDs causes a direct injection of extra free charges into the sample conduction band, promoting the formation and the subsequent radiative recombination of trions over excitons. A decreasing trend in the A-to-T intensity ratio is measured as a function of the applied voltage until the exciton band is no longer visible in the spectrum. Upon further increasing the voltage, the trion band progressively redshifts, similarly to what observed in high-pressure PL spectra above 3 GPa.^4,9,26,39^

A question then naturally arises as to how pressure can trigger the same physical process as voltage without the injection of external charges.

In wide-band-gap, n-type semiconductors, such as 1L-WS_2_, the probability of trion radiative recombination directly correlates with the n-type conductivity of the sample, which, in turn, depends on two fundamental factors: the density of donors and their activation energy.

In the present case, the application of pressure may induce the formation of some defects in the crystal, leading to a moderate increase in the donor density. This effect is irreversible and can be quantified by comparing the PL spectrum collected before and after the pressure cycle, where the exciton-to-trion area ratio (*I*_A_/*I*_T_) decreases from ∼1 to ∼0.5 (in the 1L-WS_2_/hBN case); see Figure 3b.

However, the increase in the donor density alone cannot account for the full exciton-to-trion conversion observed under pressure. Indeed, although smaller than in the pristine sample, the *I*_A_/*I*_T_ ratio measured after completing the pressure cycle is still much larger than zero, indicating that the A exciton recombination channel is restored upon pressure release.

The partial reversibility of the exciton response indicates that, besides the moderate increase in the donor density, a second, fundamental mechanism drives the high-pressure evolution of 1L-WS_2_: the decrease in the activation energy of donors.

In a reasonable scenario, the crystal compression induces a reduction in the energy distance between donor and conduction band states, leading to an increasing overlap between the corresponding wave functions; see Figure 3a. This promotes the delocalization of doping electrons into the conduction band under pressure, mimicking the extrinsic charge doping produced in gate-voltage experiments.

Under the combined increase/decrease in donor density/activation energy under pressure, the population of free electrons grows, favoring the formation of trions over excitons, until exciton recombination is completely suppressed at 3 GPa. Above that pressure, the progressive reduction in the trion energy can be regarded just as a consequence of the increase in the Fermi energy of the free electron gas due to the increase in the n-type conductivity.^4,26^

Unlike pressure-induced variation in donor density, reduction in donor activation energy is a reversible process: lattice compression modifies the interatomic bonds within the crystal lattice driving a renormalization of electronic energy levels that is reversed once the pressure on the sample is released. This explains why the A exciton recombination channel is restored after completing the pressure cycle, although with a reduced relative spectral weight compared to the pristine sample.

In summary, in our work, we demonstrate that the dominant mechanism ruling the pressure evolution of the PL spectrum in 1L-WS_2_ is the increase in the n-type conductivity driven by the moderate increase in the donor density and the progressive decrease in the donor activation energy. This process is reflected into the competition between the exciton and trion recombination channels, which ultimately drives a full exciton-to-trion conversion above 3 GPa.

Notably, the pressure-induced reduction in the activation energy of donors was experimentally observed in previous works on bulk TMDs, where the merging of n-type levels with conduction band states was found to be responsible for the onset of an *early* metallic behavior, preceding the actual closure of the band gap in the high-pressure regime.^40–42^

Our results set a reference for the interpretation of high-pressure PL measurements on low-dimensional semiconductors, in which the absence of a reliable criterium for decoupling exciton and trion contributions has often led to controversial claims on the response of the electronic band structure to the applied pressure.^32,43–47^

The phenomenology we observed parallels the exciton-to-trion conversion processes induced by the injection of extrinsic charge carriers^4,9,26,39^ or the formation of local, nonuniform strain gradients,^12–14^ while remaining mostly intrinsic and occurring at the crystal scale.

The obtained results show that high pressure can serve as a novel, powerful tool for studying exciton-to-trion conversion in 2D semiconductors, driving alternative physical mechanisms in the sample compared to previous experimental approaches. Overall, our work provides deeper insights into the excitonic properties of low-dimensional materials, encouraging further investigations in this field under high-pressure conditions.

## Supplementary Material



## Notes

Power-dependent PL spectra at different sample positions, pressure trends of the absolute intensity, power-dependent PL spectra on different substrates, 1L-WS_2_/hBN and 1L-WS_2_/diamond, details on data analysis, and reversibility of the PL spectra (PDF)

## References

[1] Chernikov A., Berkelbach T. C., Hill H. M., Rigosi A., Li Y., Aslan B., Reichman D. R., Hybertsen M. S., Heinz T. F. (2014). Exciton Binding Energy and Nonhydrogenic Rydberg Series
in Monolayer WS_2_. Phys. Rev. Lett..

[2] Sanvitto D., Hogg R. A., Shields A. J., Whittaker D. M., Simmons M. Y., Ritchie D. A., Pepper M. (2000). Rapid radiative
decay
of charged excitons. Phys. Rev. B.

[3] Sanvitto D., Pulizzi F., Shields A. J., Christianen P. C. M., Holmes S. N., Simmons M. Y., Ritchie D. A., Maan J. C., Pepper M. (2001). Observation of Charge Transport by
Negatively Charged
Excitons. Science.

[4] Mak K. F., He K., Lee C., Lee G. H., Hone J., Heinz T. F., Shan J. (2013). Tightly bound
trions in monolayer MoS_2_. Nat. Mater..

[5] Carmiggelt J. J., Borst M., van der
Sar T. (2020). Exciton-to-trion conversion as a
control mechanism for valley polarization in room-temperature monolayer
WS_2_. Sci. Rep..

[6] Jones A. M., Yu H., Ghimire N. J., Wu S., Aivazian G., Ross J. S., Zhao B., Yan J., Mandrus D. G., Xiao D., Yao W., Xu X. (2013). Optical generation
of excitonic valley coherence in
monolayer WSe_2_. Nat. Nanotechnol..

[7] Wang G., Bouet L., Lagarde D., Vidal M., Balocchi A., Amand T., Marie X., Urbaszek B. (2014). Valley dynamics probed
through charged and neutral exciton emission in monolayer WSe_2_. Phys. Rev. B.

[8] H L P., Mondal P., Bid A., Basu J. K. (2020). Electrical and Chemical
Tuning of Exciton Lifetime in Monolayer MoS_2_ for Field-Effect
Transistors. ACS Applied Nano Materials.

[9] Shang J., Shen X., Cong C., Peimyoo N., Cao B., Eginligil M., Yu T. (2015). Observation of Excitonic Fine Structure
in a 2D Transition-Metal Dichalcogenide Semiconductor. ACS Nano.

[10] Ross J. S., Wu S., Yu H., Ghimire N. J., Jones A. M., Aivazian G., Yan J., Mandrus D. G., Xiao D., Yao W., Xu X. (2013). Electrical
control of neutral and charged excitons in a monolayer semiconductor. Nat. Commun..

[11] Mouri S., Miyauchi Y., Matsuda K. (2013). Tunable Photoluminescence
of Monolayer
MoS_2_ via Chemical Doping. Nano Lett..

[12] Harats M. G., Kirchhof J. N., Qiao M., Greben K., Bolotin K. I. (2020). Dynamics
and efficient conversion of excitons to trions in non-uniformly strained
monolayer WS_2_. Nat. Photonics.

[13] Kovalchuk S., Harats M. G., López-Polín G., Kirchhof J. N., Höflich K., Bolotin K. I. (2020). Neutral and charged
excitons interplay
in non-uniformly strain-engineered WS_2_. 2D Materials.

[14] Stellino E., D’Alò B., Blundo E., Postorino P., Polimeni A. (2024). Fine-Tuning of the Excitonic Response in Monolayer
WS_2_ Domes via Coupled Pressure and Strain Variation. Nano Lett..

[15] Zeng H., Liu G.-B., Dai J., Yan Y., Zhu B., He R., Xie L., Xu S., Chen X., Yao W., Cui X. (2013). Optical signature of symmetry variations and spin-valley
coupling
in atomically thin tungsten dichalcogenides. Sci. Rep..

[16] Zhang X., Qiao X.-F., Shi W., Wu J.-B., Jiang D.-S., Tan P.-H. (2015). Phonon and Raman
scattering of two-dimensional transition
metal dichalcogenides from monolayer, multilayer to bulk material. Chem. Soc. Rev..

[17] Zhao W., Ghorannevis Z., Amara K. K., Pang J. R., Toh M., Zhang X., Kloc C., Tan P. H., Eda G. (2013). Lattice dynamics
in mono- and few-layer sheets of WS_2_ and WSe_2_. Nanoscale.

[18] O’Brien M., McEvoy N., Hanlon D., Hallam T., Coleman J. N., Duesberg G. S. (2016). Mapping of Low-Frequency Raman Modes in CVD-Grown Transition
Metal Dichalcogenides: Layer Number, Stacking Orientation and Resonant
Effects. Sci. Rep..

[19] Castellanos-Gomez A., Buscema M., Molenaar R., Singh V., Janssen L., van der Zant H. S. J., Steele G. A. (2014). Deterministic transfer of two-dimensional
materials by all-dry viscoelastic stamping. 2D Materials.

[20] Klotz S., Chervin J.-C., Munsch P., Le Marchand G. (2009). Hydrostatic
limits of 11 pressure transmitting media. J.
Phys. D: Appl. Phys..

[21] Shen G., Wang Y., Dewaele A., Wu C., Fratanduono D. E., Eggert J., Klotz S., Dziubek K. F., Loubeyre P., Fat’yanov O. V., Asimow P. D., Mashimo T., Wentzcovitch R. M. M. (2020). Toward
an international practical pressure scale: A proposal for an IPPS
ruby gauge (IPPS-Ruby2020). High Pressure Research.

[22] Segura A., Cuscó R., Taniguchi T., Watanabe K., Cassabois G., Gil B., Artús L. (2019). High-Pressure Softening of the Out-of-Plane A_2u_ (Transverse-Optic) Mode of Hexagonal Boron Nitride Induced by Dynamical
Buckling. J. Phys. Chem. C.

[23] Segura A., Cuscó R., Taniguchi T., Watanabe K., Cassabois G., Gil B., Artús L. (2019). Nonreversible Transition from the Hexagonal to Wurtzite
Phase of Boron Nitride under High Pressure: Optical Properties of
the Wurtzite Phase. J. Phys. Chem. C.

[24] Pallecchi I., Stellino E., Postorino P., Iyo A., Ogino H., Affronte M., Putti M. (2023). Experimental investigation
of electronic
interactions in collapsed and uncollapsed LaFe_2_As_2_ phases. Phys. Rev. B.

[25] Currie M., Hanbicki A. T., Kioseoglou G., Jonker B. T. (2015). Optical control
of charged exciton states in tungsten disulfide. Appl. Phys. Lett..

[26] Tran M. D., Kim J.-H., Lee Y. H. (2016). Tailoring
photoluminescence of monolayer
transition metal dichalcogenides. Curr. Appl.
Phys..

[27] Kesarwani R., Simbulan K. B., Huang T.-D., Chiang Y.-F., Yeh N.-C., Lan Y.-W., Lu T.-H. (2022). Control of trion-to-exciton conversion
in monolayer WS_2_ by orbital angular momentum of light. Sci. Adv..

[28] Sebait R., Biswas C., Song B., Seo C., Lee Y. H. (2021). Identifying
Defect-Induced Trion in Monolayer WS_2_ via Carrier Screening
Engineering. ACS Nano.

[29] Rhodes D., Chae S. H., Ribeiro-Palau R., Hone J. (2019). Disorder in van der
Waals heterostructures of 2D materials. Nat.
Mater..

[30] Golovynskyi S., Datsenko O. I., Dong D., Lin Y., Irfan I., Li B., Lin D., Qu J. (2021). Trion Binding Energy Variation on
Photoluminescence Excitation Energy and Power during Direct to Indirect
Bandgap Crossover in Monolayer and Few-Layer MoS_2_. J. Phys. Chem. C.

[31] McCreary K. M., Hanbicki A. T., Singh S., Kawakami R. K., Jernigan G. G., Ishigami M., Ng A., Brintlinger T. H., Stroud R. M., Jonker B. T. (2016). The Effect of Preparation Conditions
on Raman and Photoluminescence of Monolayer WS_2_. Sci. Rep..

[32] Han B., Li F., Li L., Huang X., Gong Y., Fu X., Gao H., Zhou Q., Cui T. (2017). Correlatively Dependent Lattice and
Electronic Structural Evolutions in Compressed Monolayer Tungsten
Disulfide. J. Phys. Chem. Lett..

[33] Oliva R., Wozniak T., Faria P. E., Dybala F., Kopaczek J., Fabian J., Scharoch P., Kudrawiec R. (2022). Strong Substrate
Strain Effects in Multilayered WS_2_ Revealed by High-Pressure
Optical Measurements. ACS Applied Materials
Interfaces.

[34] Dybala F., Polak M. P., Kopaczek J., Scharoch P., Wu K., Tongay S., Kudrawiec R. (2016). Pressure coefficients for direct
optical transitions in MoS_2_, MoSe_2_, WS_2_, and WSe_2_ crystals and semiconductor to metal transitions. Sci. Rep..

[35] Xue J., Sanchez-Yamagishi J., Bulmash D., Jacquod P., Deshpande A., Watanabe K., Taniguchi T., Jarillo-Herrero P., LeRoy B. J. (2011). Scanning tunnelling microscopy and spectroscopy of
ultra-flat graphene on hexagonal boron nitride. Nat. Mater..

[36] Dean C. R., Young A. F., Meric I., Lee C., Wang L., Sorgenfrei S., Watanabe K., Taniguchi T., Kim P., Shepard K. L., Hone J. (2010). Boron nitride substrates for high-quality
graphene electronics. Nat. Nanotechnol..

[37] Cadiz F. (2017). Excitonic Linewidth
Approaching the Homogeneous Limit in MoS_2_-Based van der
Waals Heterostructures. Physical Review X.

[38] Capeccia M., D’Alò B., Stellino E. (2024). Investigating the effects of sample-substrate
interaction in the Raman and photoluminescence spectrum of 1L-WS_2_. Il Nuovo Cimento C.

[39] Wang Z., Sebek M., Liang X., Elbanna A., Nemati A., Zhang N., Goh C. H. K., Jiang M., Pan J., Shen Z., Su X., Thanh N. T. K., Sun H., Teng J. (2023). Greatly Enhanced Resonant
Exciton-Trion Conversion in Electrically
Modulated Atomically Thin WS_2_ at Room Temperature. Adv. Mater..

[40] Stellino E., Capitani F., Ripanti F., Verseils M., Petrillo C., Dore P., Postorino P. (2022). Broadband infrared study of pressure-tunable
Fano resonance and metallization transition in 2H-MoTe_2_. Sci. Rep..

[41] Stellino E., Ripanti F., Nisini G., Capitani F., Petrillo C., Postorino P. (2021). Infrared Study of the Pressure-Induced
Isostructural
Metallic Transition in Mo_0.5_W_0.5_S_2_. J. Phys. Chem. C.

[42] Stellino E., D’Alò B., Capitani F., Verseils M., Brubach J.-B., Roy P., Nucara A., Petrillo C., Postorino P. (2023). Far-Infrared
Signatures for a Two-Step Pressure-Driven Metallization in Transition
Metal Dichalcogenides. The. J. Phys. Chem. Lett..

[43] Luo J., Li C., Liu J., Liu Y., Xiao W., Zheng R., Zheng Q., Han J., Zou T., Cheng W., Yao X., Liu Y., Zhu J. (2024). Spectroscopy
of monolayer and multilayer
tungsten disulfide under high pressure. Appl.
Phys. Lett..

[44] Nayak A. P., Pandey T., Voiry D., Liu J., Moran S. T., Sharma A., Tan C., Chen C.-H., Li L.-J., Chhowalla M., Lin J.-F., Singh A. K., Akinwande D. (2015). Pressure-Dependent
Optical and Vibrational Properties of Monolayer Molybdenum Disulfide. Nano Lett..

[45] Fu L., Wan Y., Tang N., Ding Y.-m., Gao J., Yu J., Guan H., Zhang K., Wang W., Zhang C., Shi J.-j., Wu X., Shi S.-F., Ge W., Dai L., Shen B. (2017). K-*Λ* crossover transition in
the conduction band of monolayer MoS_2_ under hydrostatic
pressure. Sci. Adv..

[46] Cheng X., Li Y., Shang J., Hu C., Ren Y., Liu M., Qi Z. (2018). Thickness-dependent phase transition and optical behavior of MoS_2_ films under high pressure. Nano Research.

[47] Dou X., Ding K., Jiang D., Sun B. (2014). Tuning and Identification
of Interband Transitions in Monolayer and Bilayer Molybdenum Disulfide
Using Hydrostatic Pressure. ACS Nano.

