# Supplementary material for: Exciton-to-Trion Conversion in Monolayer WS2 under Pressure
Source: Nano Lett. 2025 Aug 29;25(36):13496–502. doi: 10.1021/acs.nanolett.5c02823 (PMC12426993; doi:10.1021/acs.nanolett.5c02823)
Supplement: Supplementary file 1 [file nl5c02823_si_001.pdf]

Supporting Information: Exciton-to-trion conversion in monolayer WS_2 under pressure

Beatrice D'Alò,† Mattia Capeccia,† Lilia Boeri,† Paolo Postorino,† and Elena Stellino*,‡

†*Department of Physics, Sapienza University of Rome, Piazzale Aldo Moro 5, 00185 Roma*

‡*Department of Basic and Applied Sciences for Engineering, Sapienza University of Rome, Piazzale Aldo Moro 5, 00185 Roma*

E-mail: elena.stellino@uniroma1.it

S1 Power-dependent PL spectra at different sample positions

We performed pressure- and power-dependent PL measurements at four different locations on both 1L-WS_2/hBN and 1L-WS_2/diamond samples. These four points lie at the vertices of an approximately 4×4 μm^2 square area.

The main text shows the result for the point with the highest PL intensity and I_A/I_T ratio at ambient pressure. In this section, we report high-pressure, power-dependent PL spectra of 1L-WS_2/hBN and 1L-WS_2/diamond collected at the other three points: figures S1 and S2 respectively. The similar pressure evolution observed in all the measured points demonstrates the robustness of the experimental observations.

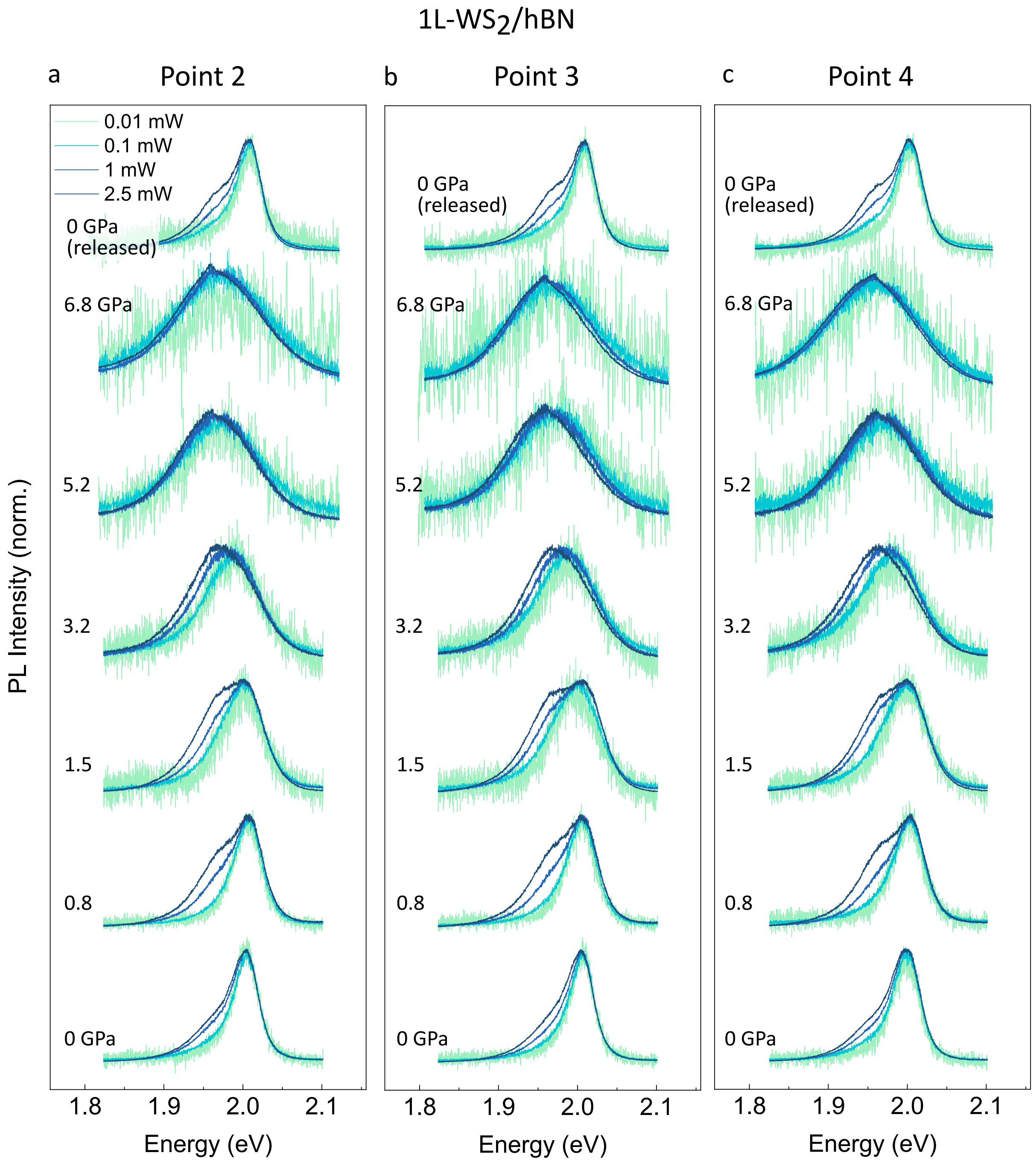

Figure S1: Power-dependent PL spectra of the 1L-WS_2/hBN sample collected at three different positions.

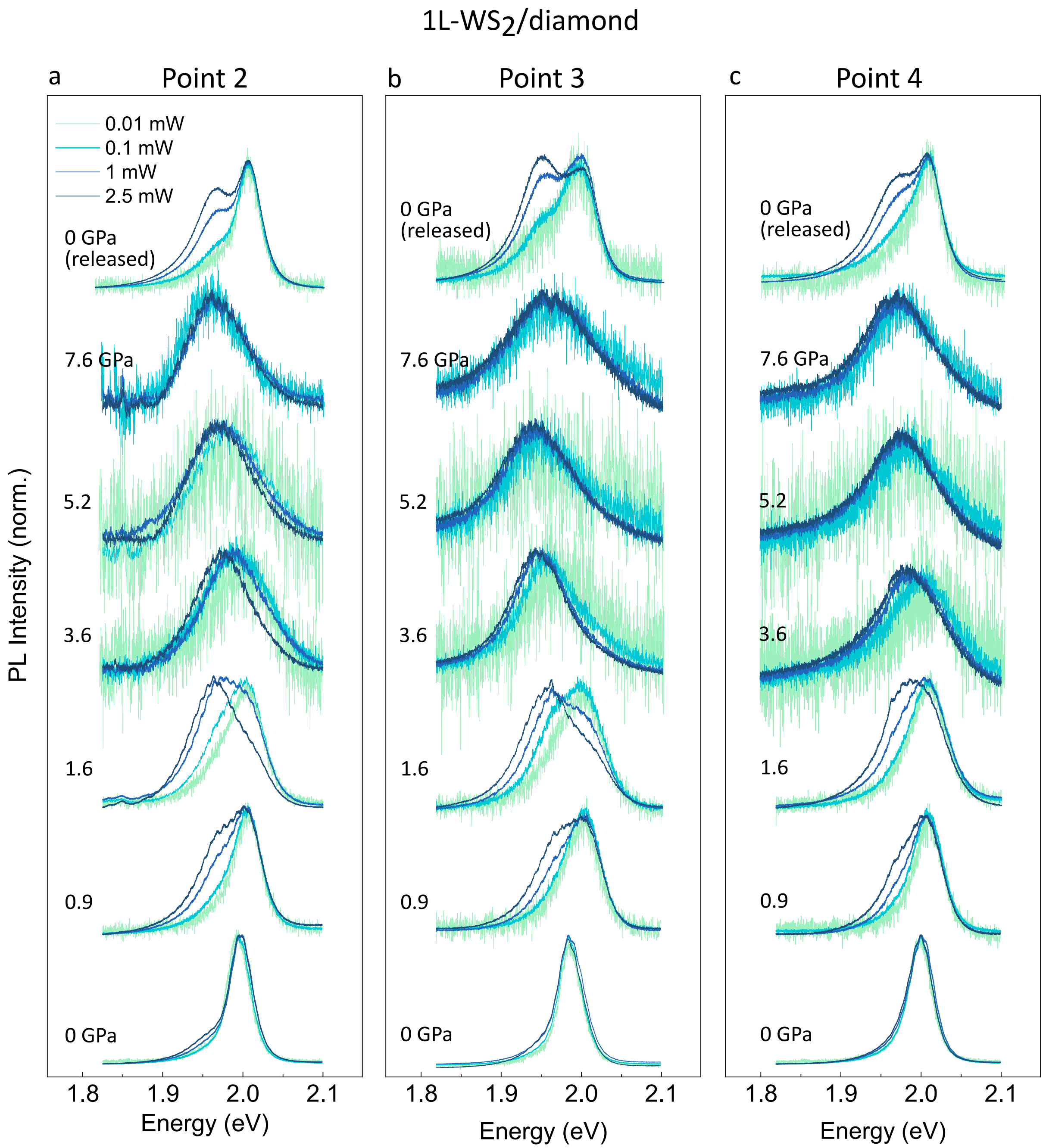

Figure S2: Power-dependent PL spectra of the 1L-WS_2/diamond sample collected at three different positions. The low signal-to-noise ratio of the 1L-WS_2 photoluminescence at 7.6 GPa and 0.01 mW prevented the measurement of the PL spectra at this pressure and laser power values.

S2 Pressure trends of the absolute intensity

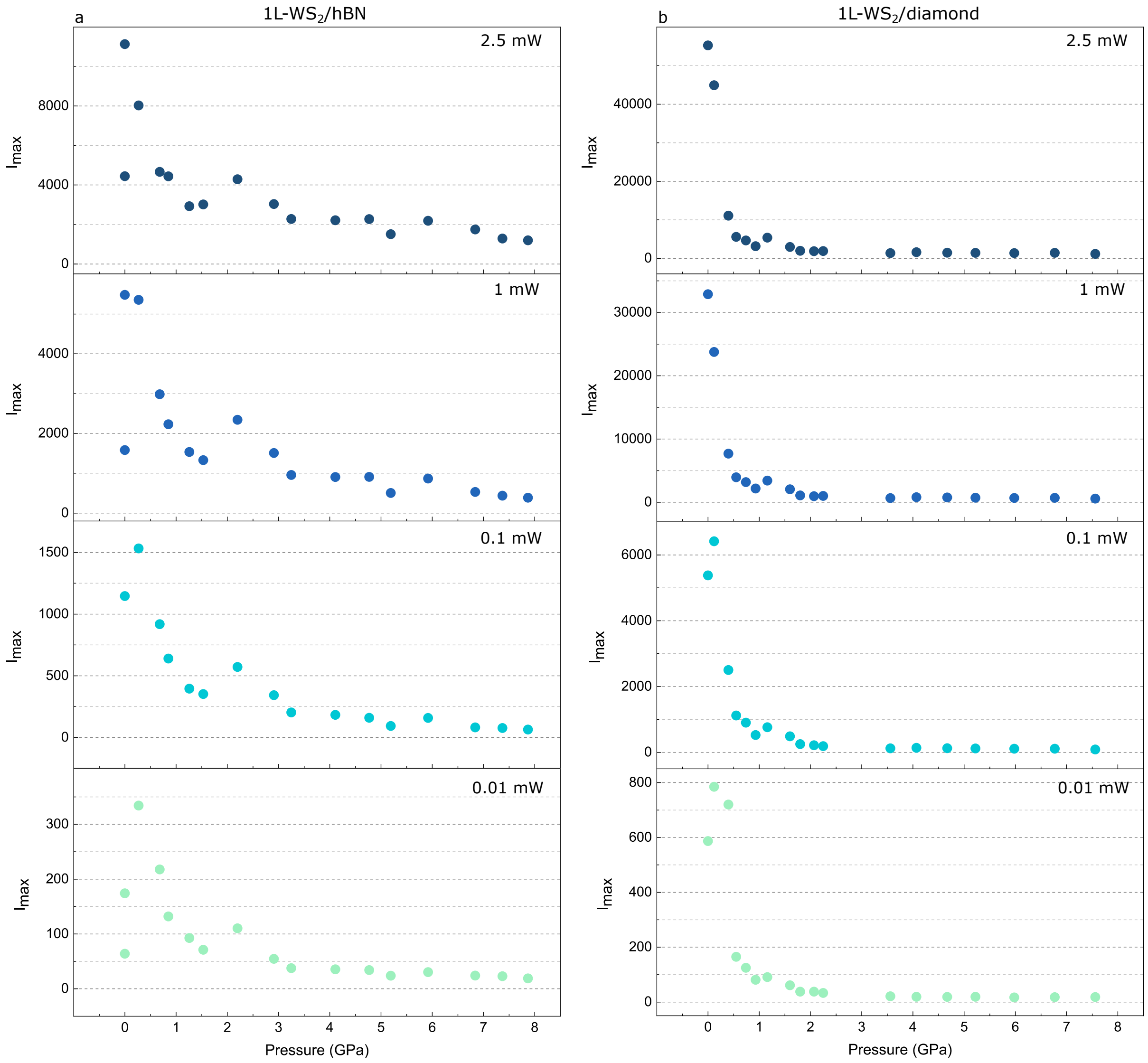

Figure S3: High-pressure evolution of the absolute intensities of the PL spectra of the 1L-WS_2/hBN (**a**) and 1L-WS_2/diamond (**b**) systems at 0.01, 0.1, 1 and 2.5 mW. The maximum value I_{max} of each PL spectrum was used to describe the PL intensity. In both samples, a rapid intensity drop occurs within the first 3 GPa, whereas at higher pressures, in the trion-dominated regime, the intensity decrease takes place at a significantly slower rate.

S3 Power-dependent PL spectra of 1L-WS_2/hBN

In this section, we report the complete data set of pressure- and power-dependent PL measurements on the 1L-WS_2/hBN sample shown in the main text, together with the results of the data analysis.

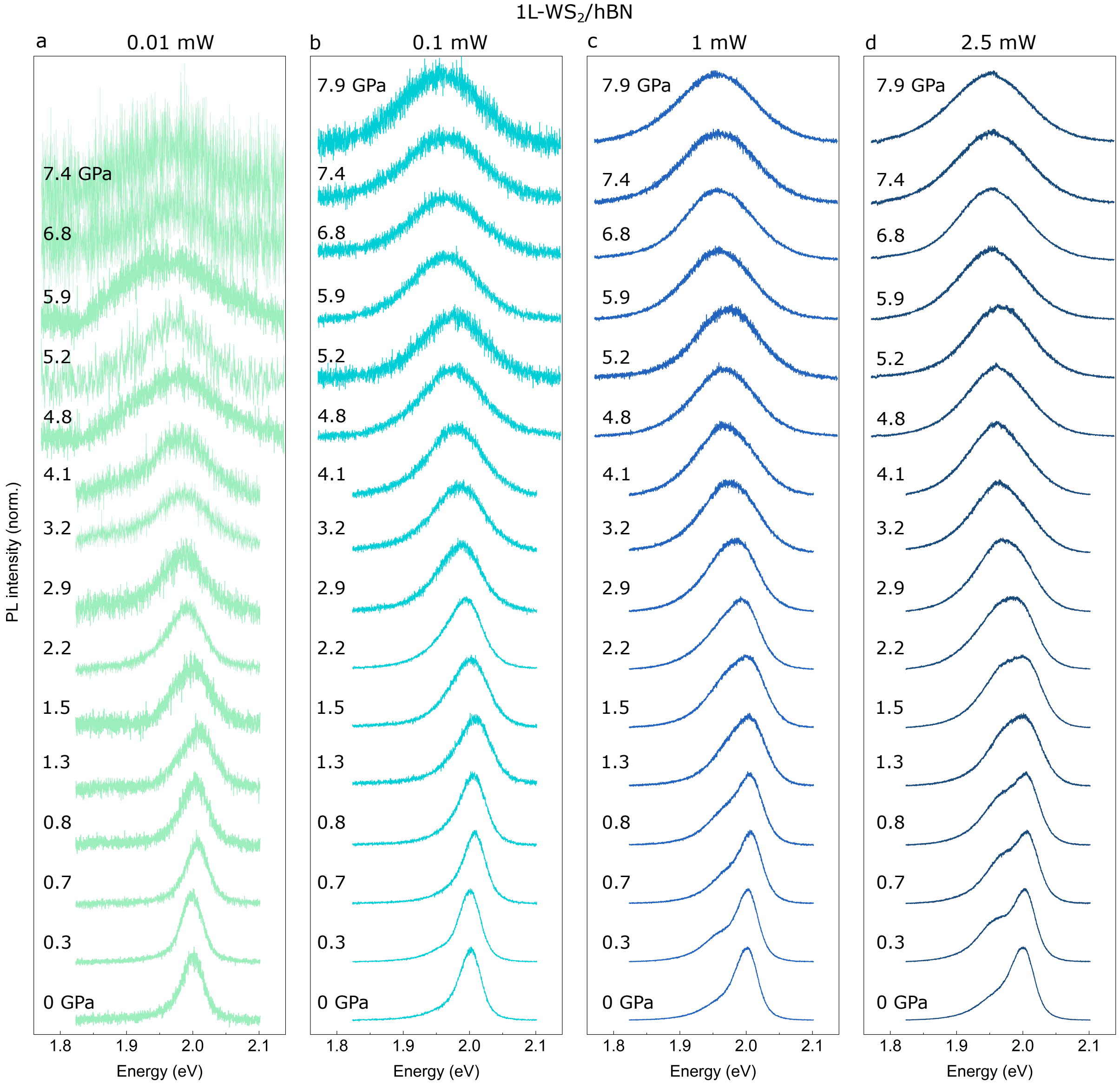

Figure S4: High-pressure PL spectra of 1L-WS_2/hBN collected at 0.01 mW (**a**), 0.1 mW (**b**), 1 mW (**c**) and 2.5 mW (**d**) of laser power.

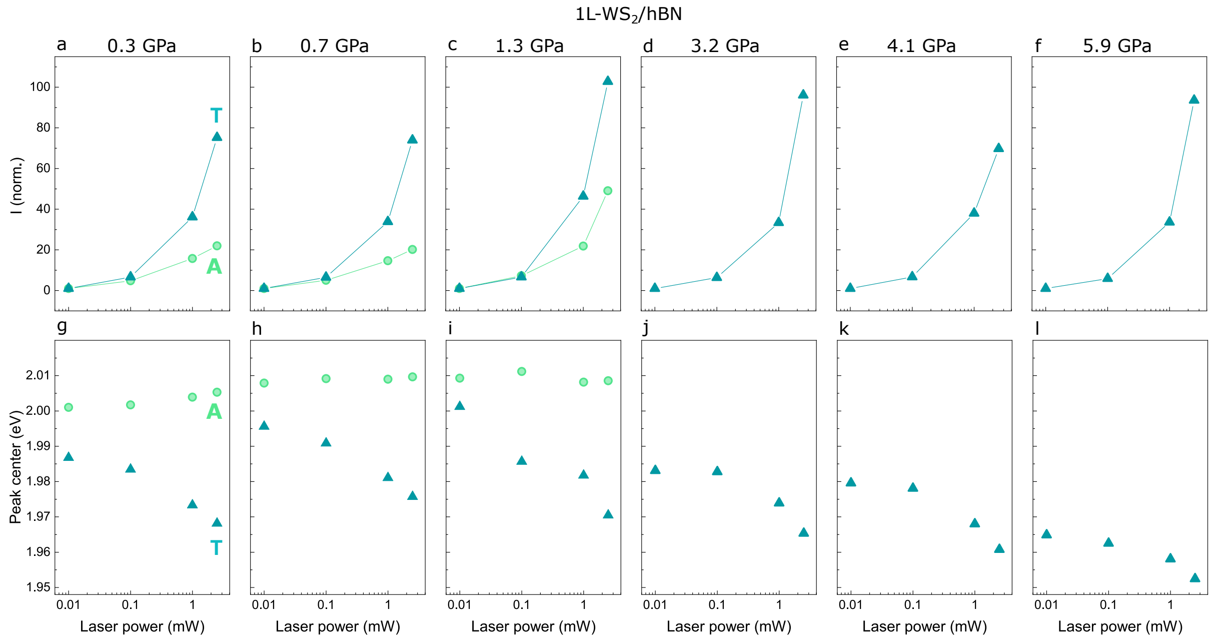

Figure S5: Power-dependent evolution of the exciton A (green dots) and trion T (blue triangles) peak parameters at different pressure values from the fit of the 1L-WS_2/hBN spectra. **(a- f)**: A and T peak intensities at increasing laser powers. The absolute intensities are normalized to the 0.01 mW value. **(g-l)**: Power-dependent evolution of the A and T peak centers at increasing laser powers. The trends of the peak intensity (**d-f**) and center (**j-l**) above 3 GPa evidence the trionic nature of the single-band PL spectra.

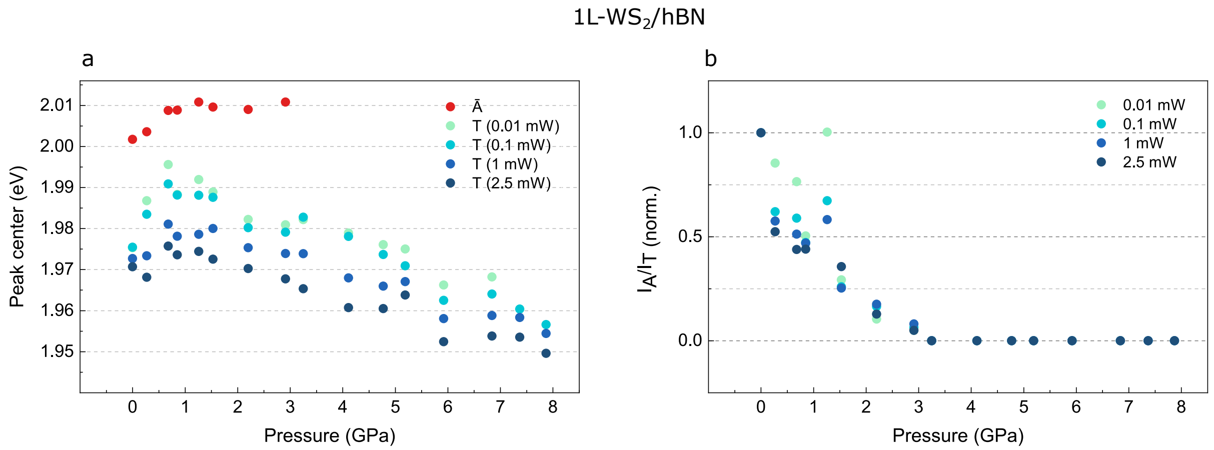

Figure S6: High-pressure evolution of the A and T peak centers (**a**) and of the exciton-to-trion intensity ratio I_A/I_T (**b**) in 1L-WS_2/hBN at different laser powers, as obtained from the fit of the data. The I_T/I_A ratios are normalized to their value at 0 GPa.

S4 Power-dependent PL spectra of 1L-WS_2/diamond

This section reports the complete data set of the pressure- and power-dependent PL measurements on the 1L-WS_2/diamond shown in the main text, together with the results from the data analysis.

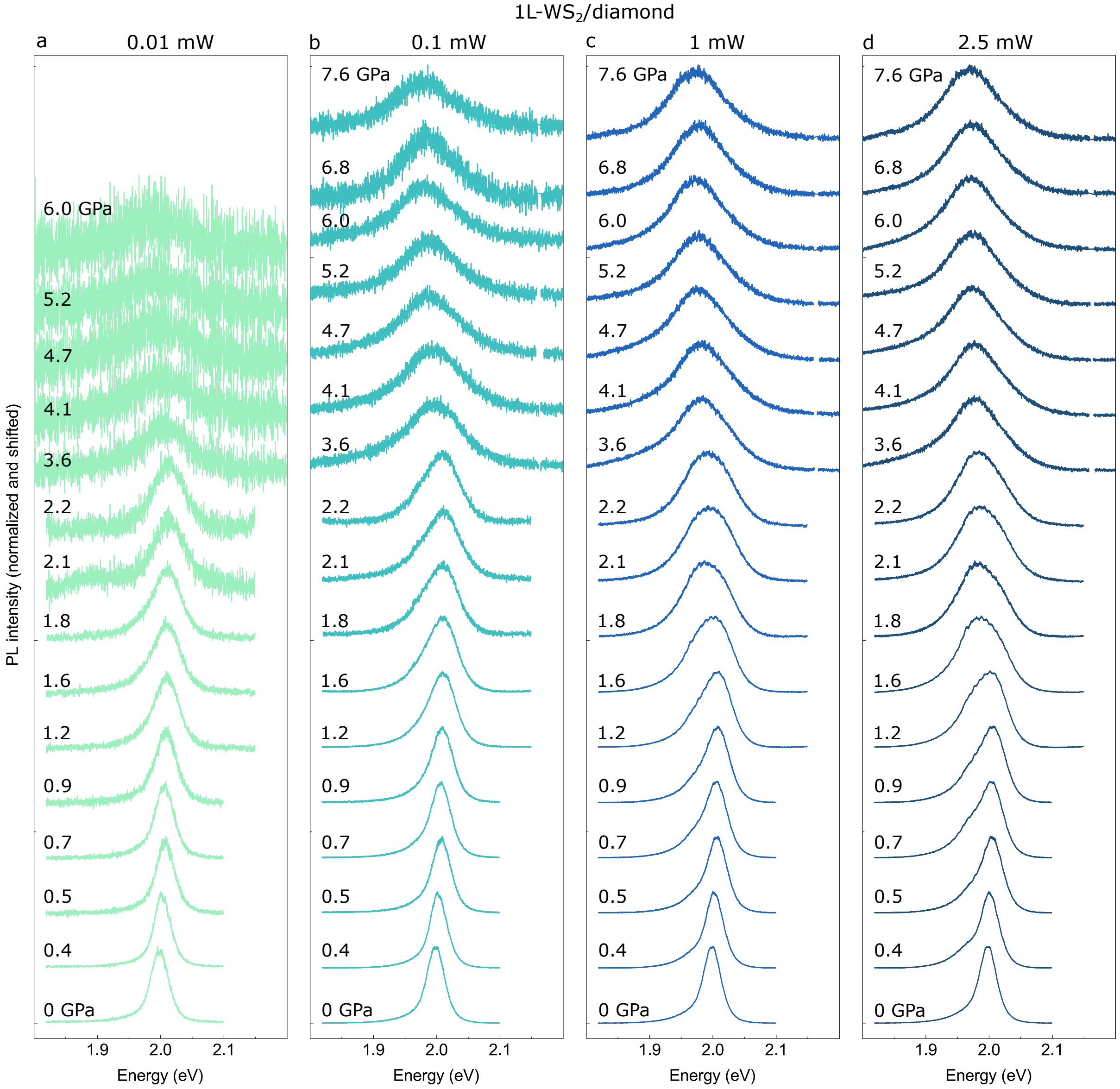

Figure S7: High-pressure PL spectra of 1L-WS_2/diamond collected at 0.01 mW (**a**), 0.1 mW (**b**), 1 mW (**c**) and 2.5 mW (**d**) of laser power.

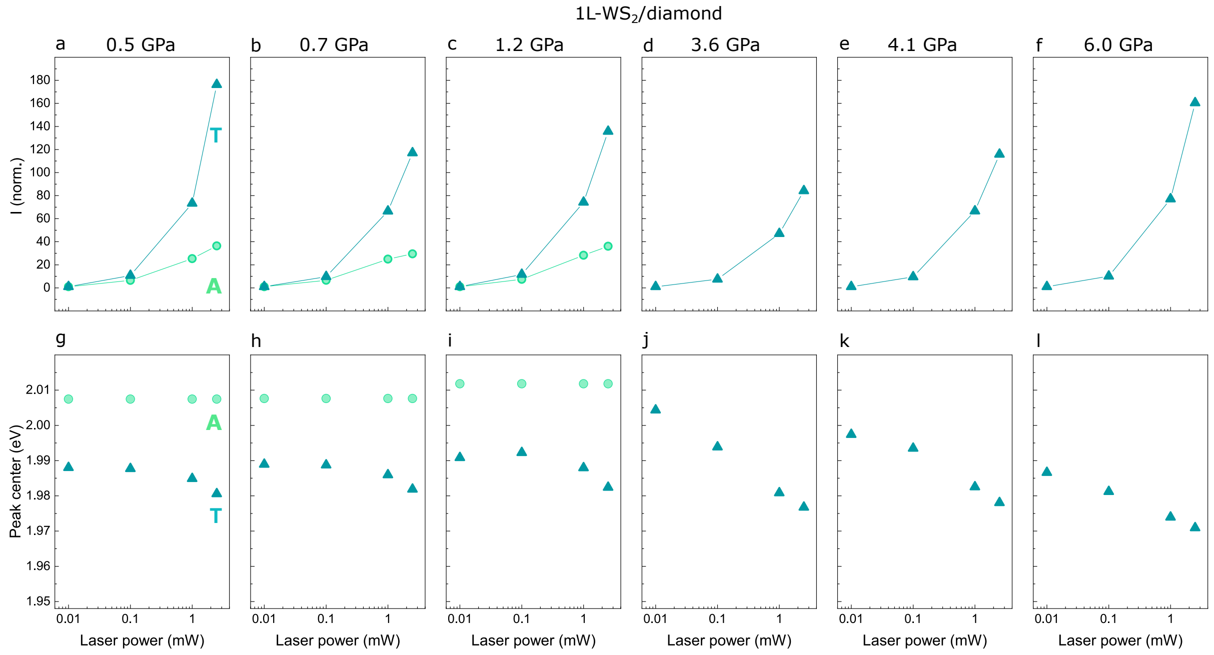

Figure S8: Power-dependent evolution of the exciton A (green dots) and trion T (blue triangles) peak parameters at different pressure values, from the fit of the 1L-WS_2/diamond spectra. **(a- f)**: A and T peak intensities at increasing laser powers. The absolute intensities are normalized to their value at 0.01 mW. **(g-l)**: Power-dependent evolution of the A and T peak centers. The trends of the peak intensity (**d-f**) and center (**j-l**) above 3 GPa evidence the trionic nature of the single-band PL spectra.

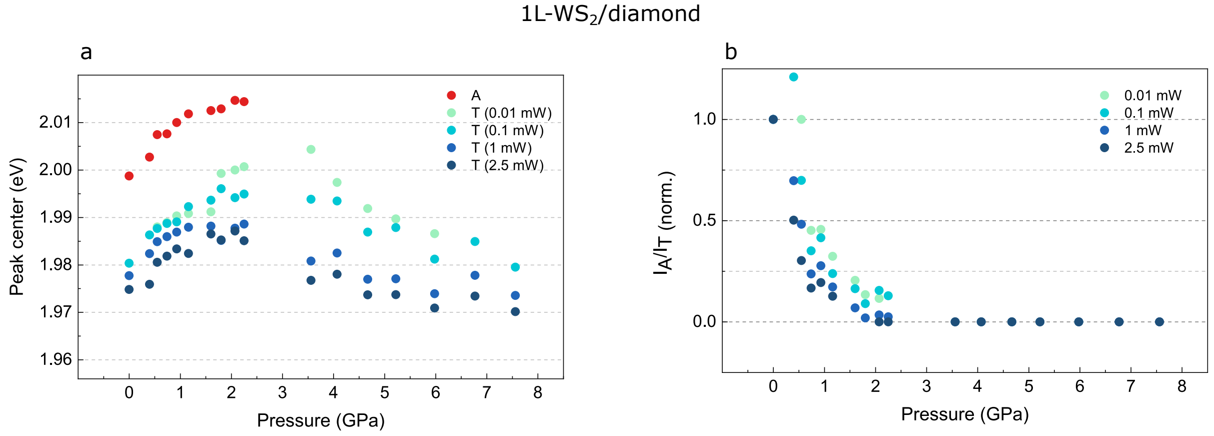

Figure S9: High-pressure evolution of the A and T peak centers (**a**) and the exciton-to-trion intensity ratio I_A/I_T (**b**) in 1L-WS_2/diamond as obtained from the fit of the data. The I_T/I_A ratios are normalized to their ambient-pressure value.

S5 Fit of the data

The PL spectra in the 0-3 GPa range are analysed by a global fit procedure that allows to account for the different power-dependent responses of exciton and trion contributions. This approach consists in the simultaneous fit of the PL spectra collected at 0.01, 0.1, 1 and 2.5 mW, at the same pressure, with two Voigtian curves. In the fit, we treat the exciton peak width, $FWHM_A$, as a shared parameter, optimizing its value simultaneously across the four PL spectra. This fitting strategy allows us to consider the width of the exciton band as constant at different laser powers,[1–3] while estimating the trion contribution even in low-intensity conditions. An example of the best-fit curves obtained by this strategy is shown in figure S10.

In the 1L-WS_2/diamond case, the smaller energy difference between the exciton and trion peaks makes it more difficult to resolve the two contributions. In this case, the global fit procedure is performed by considering as shared parameters both the exciton peak width, $FWHM_A$, and center, E_A. Through this strategy, we obtain a description of the exciton and trion bands at each pressure, avoiding over-parametrization. An example of the global fit outcome is shown in fig.S10b, while the resulting power-dependent trends of the peak intensities and energies are shown in fig.S5a-c and S5g-i at different pressure values.

The single-band PL spectra above 3 GPa are analysed by a standard fitting procedure employing a Voigt profile at each pressure and laser power value in both the 1L-WS_2/hBN and 1L-WS_2/diamond cases.

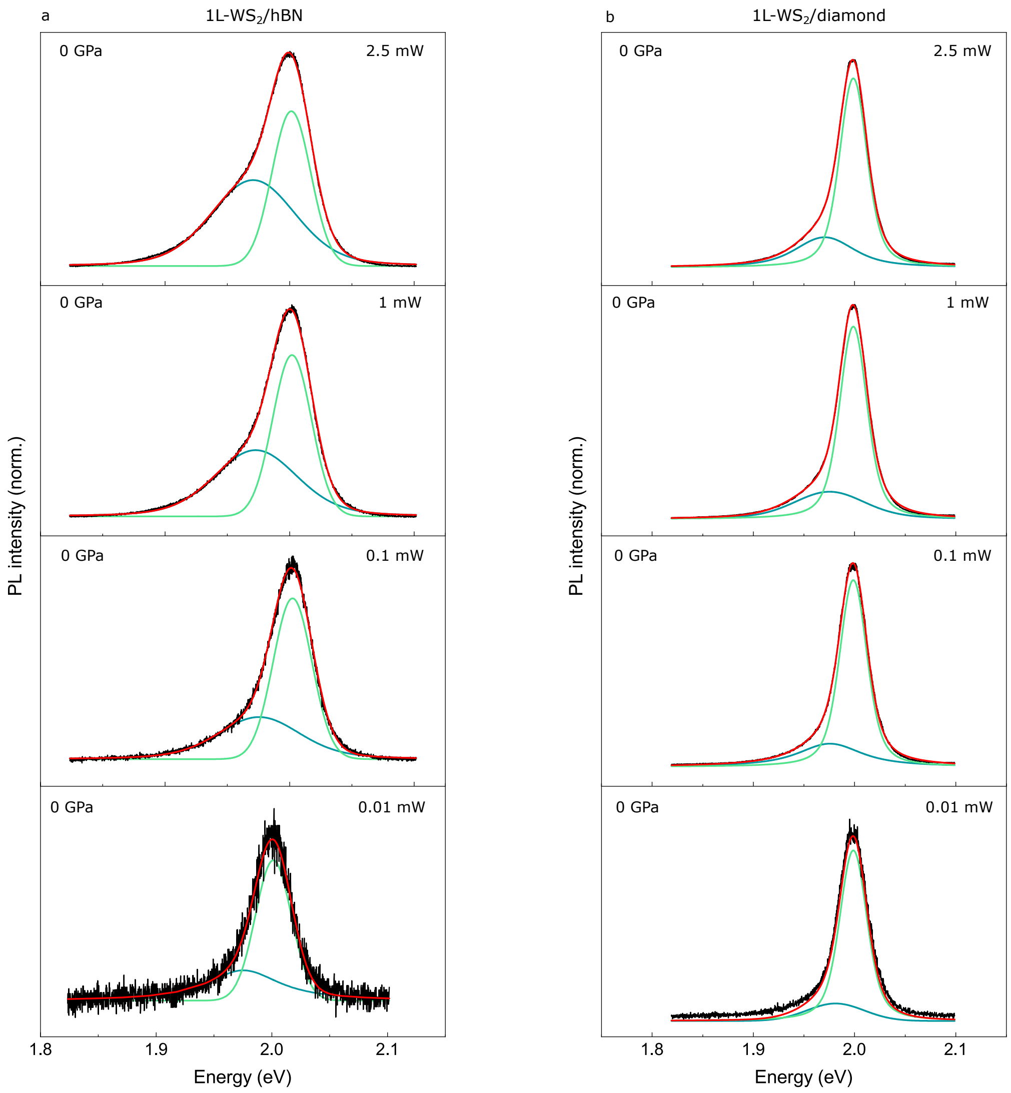

Figure S10: Best-fit curves of the exciton and trion contributions for the ambient-pressure PL spectra of 1L-WS_2/hBN **(a)** and 1L-WS_2/diamond **(b)** at 0.01, 0.1, 1 and 2.5 mW, as obtained by the global fit procedure described in this section.

S6 Reversibility of the PL spectra

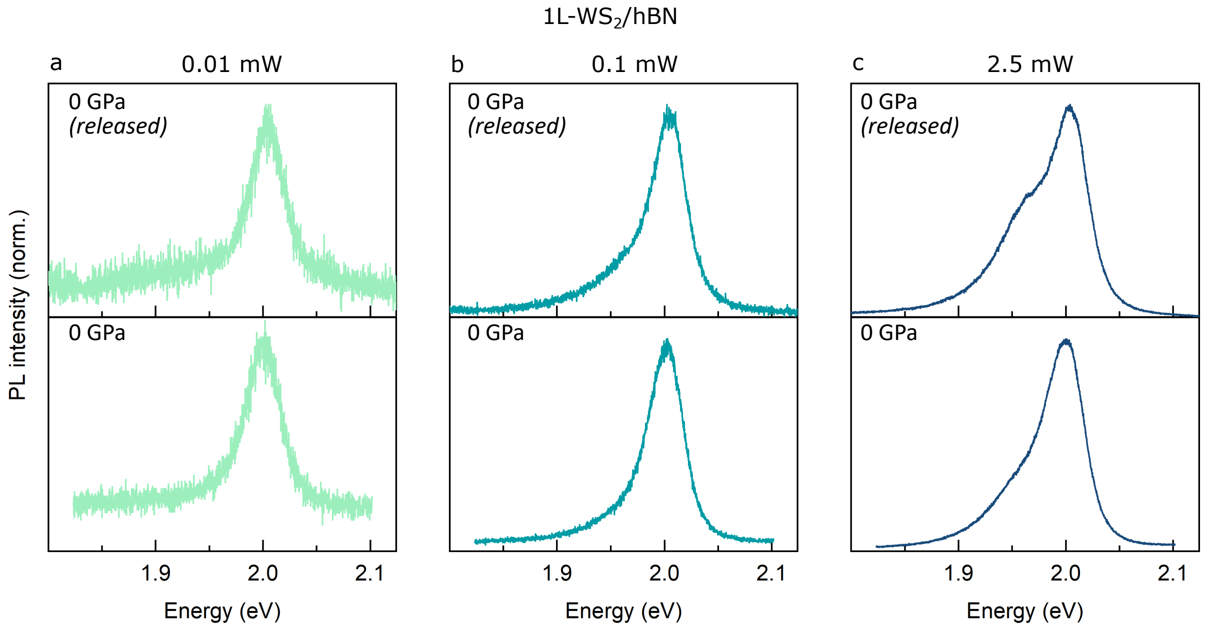

Figure S11: PL spectra of the 1L-WS_2/hBN sample shown in the main text before (*0 GPa*) and after (*0 GPa, released*) pressure application at 0.01 mW (**a**), 0.1 mW (**b**) and 2.5 mW (**c**).

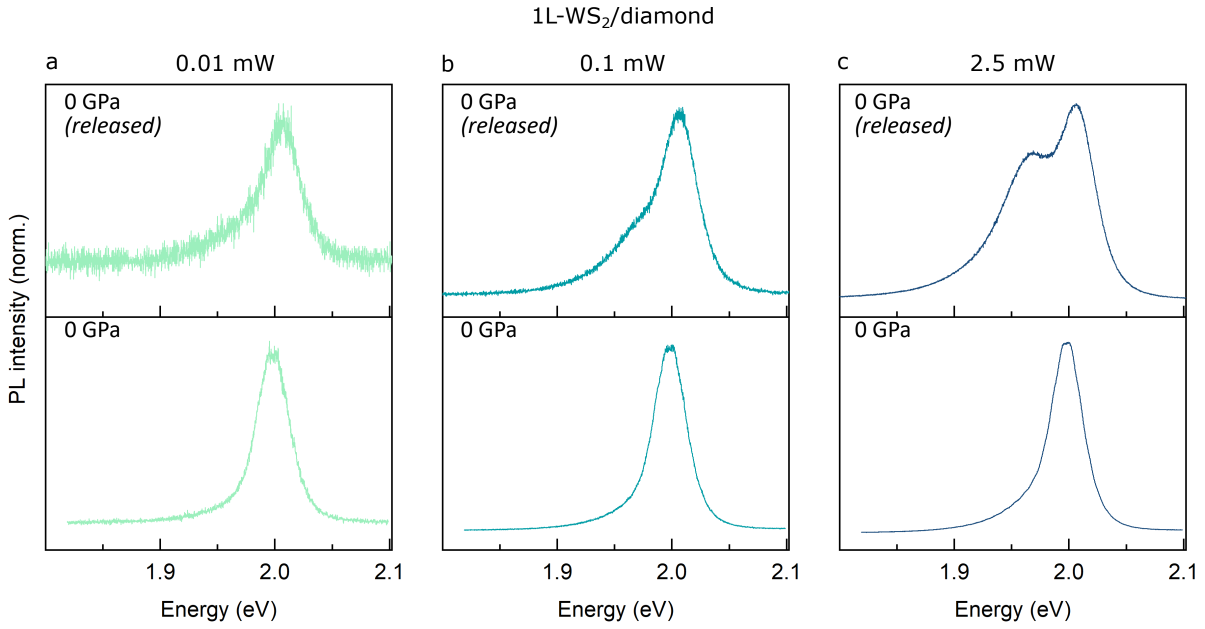

Figure S12: PL spectra of the 1L-WS_2/diamond sample in the main text before (*0 GPa*) and after (*0 GPa, released*) pressure application at 0.01 mW (**a**), 0.1 mW (**b**) and 2.5 mW (**c**).

References

(1) Tran, M. D.; Kim, J.-H.; Lee, Y. H. Tailoring photoluminescence of monolayer transition metal dichalcogenides. *Current Applied Physics* **2016**, *16*, 1159–1174.

(2) Golovynskyi, S.; Datsenko, O. I.; Dong, D.; Lin, Y.; Irfan, I.; Li, B.; Lin, D.; Qu, J. Trion Binding Energy Variation on Photoluminescence Excitation Energy and Power during Direct to Indirect Bandgap Crossover in Monolayer and Few-Layer MoS_2. *The Journal of Physical Chemistry C* **2021**, *125*, 17806–17819.

(3) McCreary, K. M.; Hanbicki, A. T.; Singh, S.; Kawakami, R. K.; Jernigan, G. G.; Ishigami, M.; Ng, A.; Brintlinger, T. H.; Stroud, R. M.; Jonker, B. T. The Effect of Preparation Conditions on Raman and Photoluminescence of Monolayer WS_2. *Scientific Reports* **2016**, *6*.
